# Creative Process and Multivariate Factors through a Creative Course “Keep Calm and Be Creative”

**DOI:** 10.3390/jintelligence11050083

**Published:** 2023-04-28

**Authors:** Aleksandra Vuichard, Marion Botella, Isabelle Capron Puozzo

**Affiliations:** 1 Education and Research Art and Technology Unit, Haute école pédagogique du canton de Vaud (HEP Vaud), 1014 Lausanne, Switzerland; 2 Laboratoire de Psychologie et Ergonomie Apliquées, Université Paris Cité, 92100 Boulogne Billancourt, France; 3 Domaine Recherche & Développement, Haute école pédagogique du Valais (HEP-VS), 1890 Saint-Maurice, Switzerland

**Keywords:** creativity, creative process diary, multivariate factors, preservice teacher training

## Abstract

Creativity has been studied for a long time and it has become a more significant topic of research in educational fields in recent decades. The present paper outlines a multivariate approach to creativity and substantiates this approach by investigating the creative process and multivariate factors through a creative course for master’s students at the University of Teacher Education in Switzerland. Our goal is to examine more specifically the stages of the creative process and the emerging multivariate factors in different creative activities. The article reports findings from the analysis of students’ creative report process diaries as well as semi-structured interviews. Drawing on experiential learning, this pilot study was conducted in collaboration with master’s student teachers (*n* = 10). The results show that the different microlevels of the creative process are the subject of variations from one creative experience to another. Most factors of the multivariate approach emerge from this kind of creative training. The discussion will allow for a review of the research results and also a better understanding of the creative process in the pedagogy of creativity.

## 1. Introduction

The current society is constantly evolving and changing. The students of today will have to solve more and more complex problems and situations. Skills such as creativity, flexibility and collaboration can serve both educators and students to find ways to innovate in their classrooms in order to develop such competencies. In the education field, creativity is particularly important in psychology. This discipline offers various definitions of creativity that promote a holistic understanding of individuals, going beyond the cognitive dimension (Guilford 1950; Sternberg and Lubart 1995, 1996; Torrance 1972).

This research draws on Lubart et al.’s (2015) multivariate approach to creativity. Creativity is defined as the ability to produce work that is both novel and appropriate. In this theoretical framework cognitive, conative, emotional and environmental factors are central to creativity. Recently Lubart proposed a new model of creativity, called the 7 C’s model. According to this model, creativity is a complex concept that can be explored through seven different facets (Lubart 2017). In this paper we focus especially on the creator’s characteristics that play a role in creativity and the creative process. More specifically, we would like to evaluate the creative process and multivariate factors of future teachers.

In his 7 C’s model, Lubart identifies the following invariants: (1) the creator (person- centered characteristics); (2) the creating (the creative process); (3) the collaboration (co-creating); (4) the contexts (environmental conditions); (5) the creation (the nature of creative work); (6) the consumption (the adoption of creative work) and (7) the curriculum (the development and enhancement of creativity) (Lubart and Thornhill-Miller 2019).

The creator encompasses individuals who actively participate in creative thinking. The creative process is defined as the succession of thoughts and actions that leads to an original and adaptive production (Lubart et al. 2015). The creative process can be described at two levels: a macro level, which presents the stages of the creative process, and a micro level, which explains the mechanisms underlying the creation of ideas, for example, divergent or convergent thinking (Botella et al. 2016). This will be analyzed in the second part of this contribution.

Regarding collaboration, Glăveanu et al. (2016) highlights the importance of social dimensions in the creativity field. He shows that distributed creativity is seen as the interaction between the individual and the world that takes place over time. The focus is on interactions with other people and the artifacts that embody group knowledge which are important contributors to the process. Aragon and Williams (2011) underscore the significance of collaborative efforts when solving problems and advocate for interdisciplinary approaches that bring together experts with diverse skill sets, rather than relying on individual efforts. Creative activity develops from the relationship between an individual and the world of work, as well as from the ties between an individual and other human beings (Fischer et al. 2005). In that sense, some authors have been interested more specifically in collaboration and its impact on creativity. Glăveanu (2010) emphasizes that the focus of research has gradually shifted to a more social paradigm—the we-paradigm—in which creativity is characterized in terms of communication, collaboration and development as a result of socialization and social interaction. The we-paradigm aims to “put the social back” into the theory of creativity (Hennessey 2003; Glăveanu 2010). The recent study of Peilloux and Botella (2016) describes both the collaboration and collective dimension of the creative process. The collective dimension refers to the process during which different individuals have to collaborate. These authors highlight the relationship between the social functioning of the group and the dynamics of the creative process.

In terms of context, the systemic model of Csikszentmihalyi (1996) suggests that creativity is the result of the interaction between the individual and the socio-cultural context. Creativity depends, therefore, on the conjunction between a domain, an environment and a person. The significance of social interaction in creative activities—both in terms of its potential and its realization in daily life, professional and eminent creativity—should not be overlooked (Kaufman and Beghetto 2009). Kaufman and Beghetto share Csikszentmihalyi’s view that creative processes and outcomes are shaped through the interactive interplay between the creator and the environment.

The traditional concepts about creativity and the ways of measuring and recognizing it in educational settings tend to emphasize creative accomplishments and traits that are static, rather than the fluid and constantly evolving nature of creative thinking and behavior (Beghetto and Corazza 2019). Recently, Corazza (2016) highlighted that creativity is a dynamic phenomenon. The path to a potentially creative outcome shifts over time and is shaped by the various social, cultural, historical and physical factors present in a particular situation. In this sense, he suggests a “dynamic” definition of creativity: “creativity requires originality and effectiveness” (p. 259) where it becomes possible for an idea to be enhanced or considered creative in a way that is delayed over time. This definition of creativity is still evolving, especially in terms of the factors that influence creativity (Capron Puozzo and Audrin 2021; Capron Puozzo et al. 2019; Vuichard and Capron Puozzo 2021).

According to the different facets of Lubart’s 7 C’s model, considering a pedagogy of creativity involves designing a school environment where the teacher allows divergent thinking and problem solving and seeks to connect learning objects with emotions. In the pedagogy of creativity, collaboration is systematically promoted allowing the broadening of horizons of possibilities, the sharing and co-construction of knowledge. In order to train creative teachers so that their learners also become creative, it is therefore imperative to work in collaboration with them to allow for the better development of creative capacities (Besançon et al. 2005). Presently, there is a new focus in the research on co-creativity (Walsh et al. 2014; Stenning et al. 2016; Schmoelz 2018) as a process that merges the individual, collaborative and communal aspects of creativity. Co-creativity is a life-wide phenomenon (Craft 2005) that is personally and internally judged (Runco 1996, 2003) instead of externally evaluated. It is collaborative (John-Steiner 2000) and communal (Chappell et al. 2012) rather than individual, and it involves conflict (Chappell 2008) and breaking away from the routine (Stenning et al. 2016) instead of being in a state of flow. However, there is still a lack of research on how students display co-creativity during classroom activities and how we can foster co-creativity in a classroom setting. Schmoelz (2018) conducted a study based on a playful pedagogical design using narrative-Socratic dialogues with teachers and students. The main findings indicate that classroom activities that incorporate playfulness offer an opportunity for co-creative reframing, meaningful dialogue, emotional expression and collaborative storytelling that is filled with actions co-determined by all participants.

Considering that each creator may involve and bring the ingredients at various points throughout the creative process (Lubart and Thornhill-Miller 2019), in the following sections we try to explain the creative process and the individual differences, taking into account multivariate factors, particularly the collaborative aspect.

## 2. Stages of the Creative Process

Cognitive and differential psychology approaches are beneficial for comprehending the creative process by considering the creator’s knowledge and personality. On the other hand, a combination of social and cognitive psychology approaches enables the exploration of the role of different factors in either promoting or hindering the creative process. For Lubart et al. (2015), the creative process is viewed as “a succession of thoughts and actions that leads to original and adapted creations” (p. 111). Since Wallas’ four-step model of creativity (1926), including preparation, incubation, illumination and verification, different studies have explored the creative process (Botella et al. 2016; Cropley and Cropley 2012; Lubart 2001; Sadler-Smith 2016). According to recent research on Wallas’ four-stage model, it has been reaffirmed that there is a lack of consensus among researchers regarding the number of stages. While Cropley and Cropley (2012) identified seven stages, Sadler-Smith (2016) based on Wallas’ book, found five stages. Various authors have suggested models of the creative process that arrange the different subprocesses involved. For example, Mumford et al. (1991) delineated a series of fundamental processes for creativity that operate on information organized in categorical structures. These processes are problem construction, information encoding, category search, specification of the best fitting categories, combination and reorganization of category information to find new solutions, idea evaluation, implementation of ideas and monitoring. The model is dynamic and allows for cycling between different processes as deemed necessary during problem solving. Indeed, this model of creative thinking processes highlights a series of new measures that could be used to assess creative potential (Mumford and McIntosh 2017). This model not only holds significance in creating novel measures for evaluating creative potential, but also suggests some new ways for developing individuals’ creative abilities. Scott et al. (2004) found that the most effective creativity training programs were those which used instruction and exercises expressly intended to develop these key creative thinking processes. Indeed, other work by Marcy and Mumford (2007) and Osburn and Mumford (2006) has shown the value of providing instruction in contributing to the more effective execution of the specific operations held to underlie the application of certain creative thinking processes. Drawing on existing creative and artistic processes, Botella and collaborators (Botella 2011) explore what the process of artistic creativity might entail. According to this more precise model, the creative process can be described at two levels: a macro level, which presents the stages of the creative process, and a micro level, which explains the mechanisms underlying the creation of ideas, for example, divergent or convergent thinking (Botella et al. 2016). Sixteen steps were held as important to investigate the artistic creative process: immersion, thinking, research, inspiration, insight, gathering, ideation (to think of new ideas), selection (to select ideas or documents), testing, precision (to refine), experimentation (to produce, to interpret), judgement (to step back, to discuss), to expound (to present one’s work to others), incubation (to let the ideas flow alone), abandonment (to renounce), and planning (to organize oneself).

Furthermore, for Botella and Lubart (2019, p. 272) “the creative process is dynamic by its components itself, their organization, their combination, the successive interactions it maintains with the environment, the unfolding nature of a phenomenon over time and its cyclical nature”. This more recent definition introduces both complexity and dynamism to the initial proposal. In his 7 C’s model, Lubart (2017) highlights that the creating or process is based on an analogical combination of more or less distant elements. The creative process as a dynamic phenomenon could be directly associated with cognitive, conative, emotional and environmental factors involved in the creative process (Botella et al. 2019). This research is based on Botella and collaborators’ (Botella 2011) 16 steps model, which was adapted to the preservice training context. This model is chosen for the self-observation of the creative process during the learning situation. Fourteen stages were proposed to evaluate a student’s creative process: problem definition, questioning, documentation, taking into account constraints, illumination, association, experimentation, evaluation, structuring, leaving it to chance, realization, finishing, pause and abandonment.

More recent studies have focused on exploring the creative process within a pedagogical context. Capron Puozzo and Botella (2018) emphasized the connection between emotions and learning by examining the creative process through the creative tasks provided in a training context with future teachers. The findings enabled the identification of the emotions experienced by the participants based on the creative activities utilized. It was found that most of the micro-processes varied during the training, and all the factors of the multivariate approach emerged. Another piece of research by Bonnardel and Didier (2016) investigated the impact of pedagogical methods on students’ creative processes and the assessment of their creative outputs. The results showed that students who underwent brainstorming-inspired training generated more ideas than their peers, while the latter proposed more constraints. Additionally, Didier et al. (2022) conducted a study that focused on verbalizing and modeling the creative process when creating technical objects. These scholars aimed to gain a better understanding of the various phases involved in developing a technical object by using observation diary of the creative process (Botella and Didier 2016). Although these different studies have explored the creative process in the educational context, no existing model comprehensively describes the interplay between all components of the creative process, the collaborative aspects in preservice teacher training and the multivariate factors. While some studies have explored one or two of these components simultaneously, there is currently no model that integrates all these elements into a cohesive framework.

In this pilot study, it is crucial to recognize the significance of investigating the creative process within a pedagogical context, particularly for the target population studied (i.e., future teachers) and the specific creative activities analyzed.

## 3. Multivariate Factors Fostering Creativity

Botella et al. (2016) suggest that the creative process can be described at two levels: the macro level explaining the stages of the creative process (such as preparation, incubation, illumination, etc.) and the micro level, which refers to the mechanisms underlying the creative process (e.g., divergent thinking, convergent thinking, associative thinking). Those types of thinking are part of the cognitive factors including all the intellectual capacities that help the development of creative thinking. The cognitive process refers to the mental ability to comprehend and assign significance to perceived information and thoughts. This human capacity for cognition is utilized in the creative process, which involves activating subsidiary faculties and mechanisms for generating and organizing novel ideas or building upon existing ones. It should be emphasized that an individual’s personal attributes can offer diverse levels of creative potential, contingent on the specific task or area of expertise. Additionally, the notion of cognitive style is frequently discussed, referring to the distinct ways in which an individual perceives, recalls, processes, assimilates, transforms and applies information. This style of information processing is intricately linked to an individual’s learning style (Guerra and Villa 2019). Concerning the conative factors, Lubart et al. (2015) explain that they bring together all the behaviors that are part of an individual’s habits, such as motivation, perseverance, openness to new ideas, risk taking, etc. For example, Lubart and Thornhill-Miller (2019) have demonstrated that some studies indicate that individuals’ inclination towards taking risks may differ depending on the specific domain of activity. To illustrate, an individual may exhibit a willingness to take a risk in a sporting context and endeavor to execute a new technique while ice-skating in a competition yet may not demonstrate the same level of openness to experimentation when engaging in a creative pursuit, such as the visual arts. Similarly, another person may display an eagerness to invest their efforts into a novel business concept but may not feel comfor2presenting new ideas in a written task (Lubart and Thornhill-Miller 2019). Both positive and negative emotions play an important role in the creative process (Audrin et al. 2020; Botella 2011). According to Davis (2009), a positive mood state can boost productivity in divergent thinking, possibly because it leads to a more relaxed standard for assessing the merit of an idea. Moreover, a study by Peilloux and Botella (2016) shows a specific affective profile for each stage of the creative process. These authors noticed that the stages that mostly occur at the beginning of the process, such as immersion, thinking or research, are associated with positive effects. On the other side, the stages that occur mostly at the end, such as judgement or exposition, are associated with negative emotions. However, these affective profiles can also be explained by the nature of the task. For example, a task that is perceived as challenging or novel may elicit a positive-activation affective profile in individuals, characterized by high levels of positive affect and arousal. In contrast, a task that is perceived as uninteresting may lead to a low-activation affective profile, characterized by low levels of both positive and negative affect.

Finally, environmental factors are characterized by all situations and interactions, including professional, family, school, social, cultural, national, local, etc. environments. They can be decisive in helping to reveal, activate and develop creative capacities. For Lubart and Thornhill-Miller (2019) the creative context is comprised of both physical and social spheres. Thus, the context refers to the favorable or unfavorable conditions for the development of creativity and is linked to environmental conditions. Some authors demonstrate that creativity and creative performance are influenced by the work environment (Dul et al. 2011; Amabile 1996; Amabile et al. 1996; Amabile and Pratt 2016). In this direction, Amabile initiated her research on the relationship between work environment, organizational factors and creativity, i.e., organizational creativity, combining for the first time managerial and psychological aspects. School can also be viewed as an organization which can benefit greatly from a creative environment. In the school context, Besançon et al. (2005) point out that the diversity of cultural activities as well as family structuring, which are environmental parameters, have a direct influence on creativity and the construction of creative thinking in children.

In this paper, our focus is centered on the social and collaborative dimensions, as well as on the environmental-social factors of students’ creative processes in experienced creative activities.

## 4. From Social Creativity to Collaborative and Collective Creativity

In the late 1970s, more psychologists started to understand that creativity takes place in a social context (e.g., Harrington 1999). With this in mind, Simonton (2000) demonstrated that creativity is greatly influenced by social factors. He also showed that there was a change among psychologists in recognizing that creativity is not an individualistic phenomenon, but rather occurs within a social context. This recognition was further developed in the 1980s, when a distinct social psychology of creativity was established to complement the existing cognitive, differential and developmental perspectives on creativity. As evidence of this trend, scholars such as Harrington (1999), Amabile (1983) and Westmeyer (1998) began to publish works that explicitly explored the social aspects of creativity. For Westmeyer (1998), the creativity of a product is determined by evaluators or judges who possess expertise in certain production domains. The social context in which the evaluation takes place also plays a role, and the passage of time may affect the evaluation. Moreover, Amabile (1996) investigates the role of social factors such as the cultural context, the dynamics of a group or team, etc. in the creative process. She argues that the social environment can have a significant impact on an individual’s creativity. For example, supportive and positive social environments can enhance creativity by giving individuals the freedom to explore and experiment with new ideas without fear of criticism or negative judgement. Conversely, negative social environments, such as those that are highly competitive or critical, can inhibit creativity by creating an atmosphere of fear and anxiety that limits an individual’s ability to think and create freely.

In the process of transition from an exclusively individual psychology of creativity to a more socially orientated one, three main concepts emerged: social creativity, collaborative creativity (Paulus et al. 2012) and collective creativity (Fischer and Vassen 2011; Pundt 2021). In order to better understand the social dimension of creativity, we will define these terms.

Social creativity is a large topic in which different forms such as collaborative and collective creativity can be discussed. For Fischer et al. (2005, p. 483), social creativity is the product of different forces: “the individual; the mix among individuals (the distinctive interests, skills and knowledge that compose specific communities); and the interactions between them and their social and technical environment”. Referring to social creativity, people collaborate with each other by taking up tasks that fit well with their knowledge and personal interests (Fischer et al. 2005). For Purser and Montuori (2000), the term social creativity emerged by showing that creativity is the result of human interaction and collaboration and demonstrating a renewed interest in group creativity (Nemeth and Ormiston 2007; Paulus and Nijstad 2003 cited in Glăveanu 2010). Research in the field of social problem solving, as demonstrated by the works of Dodge (1986) and Spivack and Shure (1974), has emphasized the importance of creative abilities in the development of social skills. Mouchiroud and Bernoussi (2008) conducted a study focusing on the type of creativity that can be expressed in solving social problems. The results show that social creativity performance is linked to socially relevant variables, such as social competencies, popularity and parenting style. They indicate that the capacity for social creativity, as evaluated through divergent thinking tasks, is associated with a range of individual and experiential factors that are all related to how well an individual adapts to their social environment.

The term collaboration seems to be a significant element in social creativity. Glăveanu (2011, 2021) refers to group or team creativity as collaborative creativity. He makes a distinction between the socio-cognitive and socio-cultural aspects of this topic. The socio-cognitivists tend to isolate the impact on others of some variables measured at the level of the individual only, while the socio-culturalists think that the social and the individual dimensions are linked by the act of collaboration (Glăveanu et al. 2021).Collaborative creativity does not describe only instances of social interaction in groups or organizations, but equally applies to individual creative processes. For Lubart and Thornhill-Miller (2019), collaboration refers to the interactions in which two or more individuals participate in a joint development, often with different or complementary skills, often creating something that they could not or would not create on their own. Some creativity is simply more easily recognized and labeled as “collaborative” because of its proximity in time or space to the others that helped make it happen. Thus, creativity is more of an individual act in some societies, whereas it is inherently more collaborative in others. In that sense, creative thinking cannot be isolated from the cultural matrix that promotes it and is fundamentally formed by it (Glăveanu et al. 2019).

Some authors mention a collective creativity (Sawyer 2012; Pundt 2021). Developed on the basis of biographical information, Pundt (2021) suggests a unique model of collective creativity. Although the model focuses on collective musical creativity, the model itself can be applied to other areas. The model proposed by this author includes an interaction between individual creativity focused on inspiration, elaboration and evaluation, and team or collective creativity that involves the same processes. Not surprisingly, the process of communication figures significantly in this model as the mechanism by which individual creativity will manifest itself as collective creativity. Sawyer (2012) also highlights that historical research confirms the increase in collaboration. Primarily, this is due to the growth in the complexity of the problems to be solved in research and in other fields. This is why collective creativity is more important than individual creativity, because it incorporates more points of view and expertise (Sawyer 2012). For this same author, a group is more creative than isolated individuals, provided that the team members have been working together for some time, but especially when they share certain conventions and have common knowledge.

Creativity as a collective phenomenon, therefore, implies dynamics of collaboration and even cooperation. In the pedagogical field, the principal difference between cooperative and collaborative learning is that the former is based on the principle of interdependence and the latter on sharing and bringing together knowledge (Baudrit 2005). According to the Johnson and Johnson model (2000), cooperative learning is instruction that involves students working in teams to accomplish a common goal by optimizing the learning of each person. Moreover, working cooperatively helps students to develop their social skills and take control of their learning. These authors underline that students in cooperative groups can achieve their learning goals if, and only if, the other students with whom they are cooperatively associated achieve theirs. One of the most significant conditions of cooperation is the fact that members of a work team must perceive that their success is conditional on the success of other team members in achieving a common goal (Johnson and Johnson 1989, 2009).

## 5. Objectives and Hypotheses

Creative processes can differ in terms of the number of stages involved and the characteristics of each stage. The aim of the present study is to directly question student teachers on the stages of the creative process based on Botella and collaborators’ model (Botella 2011). Our goal in this paper is:

1. to identify the stages and multivariate factors that emerge in students’ creative processes by analyzing the differences between the different creative activities;
2. to describe the importance of collaborative aspects for creativity in the specific context of training.

We hypothesized that:

(1) Most of the stages emerge in the students’ creative processes.
(2) Different multivariate factors emerge during the creative process.

To be specific, students do not experience the same stages of the creative process during every creative activity. In order to test these hypotheses, we conducted a study which evaluated students’ creative processes and the aforementioned factors.

## 6. Method

This research design drew on action research (Barbier 1996; Van der Maren 2003), in which professors and students are involved in the process as collaborative actors. Furthermore, this course is based on experiential learning (Mandeville 1998, 2001) which allows students to live creative experiences from which they are then invited to make connections with the theoretical contents of the course (Capron Puozzo and Botella 2018). The aims of this interdisciplinary elective course are not only to train in creativity and its pedagogy, but also to improve the transversal capacity of students’ creativity by bringing together multiple disciplines, such as history, art, science, design thinking techniques, etc.

### 6.1. Participants

This research was conducted at the University of Teacher Education in Switzerland in the fall semester 2018–2019. Participants in the research comprised student secondary school teachers (*n* = 10) on a master’s degree course (at the beginning of the program). This interdisciplinary course included future teachers of different disciplines such as mathematics, French, physical education, science, etc. Three students were female and seven were male (mean age = 28.6; sd = 2.22; span = 23–31). The students voluntarily participated in this elective course and provided their informed consent. We provided clear and concise instructions that explained the purpose of the study and the response format.

### 6.2. Material

#### 6.2.1. Evaluation of the Creative Process through the Creative Process Report Diary (CRD)

The creative process report diary (CRD) is a relevant analytical tool for evaluating a student’s creative process and the multivariate factors involved. Thus, the CRDs are used in order to respect an ecological approach to the creativity process and are particularly used when a study is conducted outside the laboratory (Botella 2011). The adapted diary is designed to collect both quantitative and qualitative data to track the process experienced in terms of micro levels (Botella et al. 2016) and learning.

The quantitative element is based on the closed questions of a CRD (Botella et al. 2011; Botella et al. 2019) in order to understand the creative process experienced by future teachers and the multivariate factors involved. This element was collected immediately after the training. The students had to:

1. Check off one of the 14 stages of the creative process in which they found themselves during the creative activity: problem definition, questioning, documentation, taking into account constraints, illumination, association, experimentation, evaluation, structuring, leaving it to chance, realization, finishing, pause and abandonment. The students were aware that they could use none, one or many stages at each evaluation.
2. Indicate on a five-point Likert scale the degree to which a list of multivariate factors was mobilized. These are cognitive and conative factors (perseverance, discipline, patience, perfectionism, strength, getting organized, concentration, decision-making, quality, dynamics); environmental-social factors (discussion, listening, collaboration, implication and being friendly) and emotional factors (curiosity, boredom, confusion, surprise, anxiety, frustration, enthusiasm, disappointment, awakening, pride, hesitation, inspiration, satisfaction, stress, exhaustion), including epistemic emotions (curiosity/interest, boredom, confusion, surprise, anxiety, frustration, enthusiasm). For the emotional factors, we focussed on seven of the nine training techniques. Indeed, the questionnaire took into account the emotions of the group and only the first seven creativity techniques were carried out collectively. The last two courses, conducted individually, are not considered in this section.

#### 6.2.2. Interview Guide

The second tool used for the qualitative element one and a half years after the training was a semi-structured interview based on evocation (Vermersch 2011; Aden 2016). The complementary interview method was conducted in order to better understand what persisted from this creativity course and whether it had an impact on the students’ teaching practices in the long term. The students were interviewed at the end of their master’s degree. We chose this methodological approach because it allowed us to have access to the students’ perception, that is the narration of the links they make between the training and their practices. This type of interview allowed us to see the students’ representations in terms of creativity and training. By reliving the past situation, the student can analyze his or her practice and bring to his or her consciousness what he or she did not know at the time. Specifically, the goal was to understand whether the creativity course and its pedagogy had: (a) contributed to their perceptions in terms of creative training; (b) had an impact on interpersonal relationships; (c) contributed to their professional development in terms of changes in their teaching practices and (d) had an impact on their emotions. We therefore developed a grid (Ghiglione and Matalon 1992). We determined the categories that we used to score the interview data. These categories are based on the research question and the data collected during the interview. In the interviews, we asked questions about their professional and skills development, the long-term impact of the creative course on students and any remarkable moments of creative activities. In this paper, we are interested in the social and collaborative aspects mentioned by students.

#### 6.2.3. Procedure

This creative course “Keep calm and be creative” covers three levels. The first level is theoretical and refers to the learning of content concerning creativity and its pedagogy. The second level is pragmatic, as it deals with the didactic transposition of theoretical content. The objective is for students to transpose these elements to learning situations in a school context. Finally, the third level focuses on experiential learning (Mandeville 1998, 2001), which allows students to experience and experiment with creative techniques. Scott et al. (2004) conducted a study through the quantitative meta-analysis of program evaluation efforts, drawing on 70 prior studies. The findings indicate that well-designed creativity training programs generally lead to improved performance, with these effects observed across different criteria, settings and target populations. Factors contributing to the effectiveness of these training programs were also examined, revealing that programs which focus on the development of cognitive skills and heuristics, using realistic exercises appropriate to the relevant domain, tend to be more successful. Our creative course shares a common foundation with other creativity programs, such as divergent thinking, problem solving and performance. In our research, we also focus on a cognitive, social, personality and motivational framework in the design of course content.

After having lived this creative experience, the students are then encouraged to reflect on their experience, in relation to the theoretical content of the course, through the CRD (Botella et al. 2011).

At the end of each creative activity students filled in their course diaries in order to keep a record of their experiences throughout the semester. Nine creative activities were experienced. Seven of the nine activities (described in Table 1 below) were carried out collectively promoting collaboration, exchange and group well-being. Two of them were conducted individually.

The quantitative data from the questionnaires were analyzed using SPSS (IBM SPSS Statistics, 26) software while NVIVO (NVivo, 12) was used for the qualitative data analysis.

## 7. Results

### 7.1. Quantitative Data: Creative Process

In order to define and analyze the stages of the students’ creative processes, we used Friedman’s non-parametric ANOVA (repeated measures) to test the variation between nine activities. This result indicated that the medians of each of the nine activities were not equivalent. Significant variations were present in these stages: problem definition, questioning, illumination, evaluation and finishing.

Moreover, after ANOVA, we conducted post-hoc analyses using the Durbin–Conover test to examine the pairwise differences between activities. The results of five significant stages are presented in Table 2. We chose the standard threshold of a *p*-value below .05 to assess the significance of our results.

We found that the test was significant for problem definition (Chi^2^ (8) = 16.6, *p* =.035). The definition served to discover the theme and to focus on the subject. This stage corresponds to the preparation stage in Wallas’ model. The results indicated that during the world café, creative environment and museum activities and the escape game many participants checked the definition stage, which was not the case for the marshmallow challenge or the crea-experience. For the questioning stage (Chi^2^ (8) = 21.8, *p* = .005), we observed that in the marshmallow challenge, the creacapture, world café, land art, creative environment, and museum activities, the escape game and the Bionique activity this stage was chosen by most students while for the crea-experience this phase was not selected by many participants.

The test was also significant for the illumination stage (Chi^2^ (8) = 20.4, *p* = .009), we found that in activities such as the creacapture, world café, land art, creative environment and museum activities this stage was more present than in the crea-experience. Moreover, the test was significant for the evaluation stage (Chi^2^ (8) = 22.2, *p* = .005); in the marshmallow challenge, the creacapture, world café and museum activities, the escape game and the Bionique activity most students were in this stage and were not in the crea-experience. For the finishing stage the test was also significant (Chi^2^ (8) = 16.9, *p* = .032), indicating that in the Bionique activity there were not many students noting this stage but in the world café, land art, creative environment and museum activities a lot of them checked this stage. Finally, this test was not significant for documentation, taking into account the fact that constraints, association, experimentation, structuring, leaving it to chance, realization, break and abandon were not significant. It also meant that all these stages did not differ between activities.

In order to identify the stages of the creative process and to test our first hypothesis we observed the frequencies of each of the stages during the nine training creative activities. In particular, we analyzed the percentage of participants who checked a level in the process at the end of each creative activity experienced during the course (Table 3). Looking at Table 3, we can see that most stages oscillate between the various activities. For example, the students demonstrated a marked interest in questioning and evaluation in the marshmallow challenge which was carried out with the intention of being an ice breaker. The second activity, creacapture, asking students to represent creativity via a video, image, recording, etc., involved problem definition, questioning and evaluation. During the creativity marathon, students participated in two different creativity activities: the world café and land art acitivities. For the world café, they mostly reported definition, questioning, illumination, evaluation and finishing. The students also participated in an artistic activity, the land art activity and had to create a work/piece with materials from nature. In this activity the majority of the levels were present: problem definition, questioning, illumination and finishing. In the creative environment where students used the ordinary and reverse brainstorming technique, all participants experienced the following levels: problem definition, questioning, illumination and evaluation. The museum activity and escape game were both characterized by problem definition, questioning, illumination and evaluation. On the other hand, the crea-experience activity did not mobilize these stages. The last creativity technique called Bionics mobilized the students in terms of problem definition and evaluation.

### 7.2. Quantitative Data: Multivariate Factors

In this section we look at the multivariate factors and try to find out their intensity.

#### 7.2.1. Cognitive and Conative Factors

Significant variations were present for the following cognitive, conative and environmental factors: patience, discipline, getting organized, decision-making, dynamics, collaboration and implication.

In order to test our second hypothesis, we noted that among the cognitive and conative factors, patience was the most used factor during the land art activity (M = 4.7), where students had to create a work together from the materials found in nature. In addition, discipline increased with the escape game (M = 4.7), where students were invited to collaborate in order to solve enigmas related to the theoretical content. The participants gave higher scores for organization during the land art activity (M = 4.8). On the other hand, the marshmallow challenge (M = 3.11) did not score highly for this factor. For the decision factor we observed the highest score in the museum activity (M = 4.67) and the lowest in the marshmallow challenge (M = 2.67). During the world café and land art activities, the dynamic factor increased (M = 4.70). Regarding environmental factors, the Table 4 shows that implication was the most relevant in the creative environment (M = 4.88) and museum activities (M = 4.78) in the techniques of idea generation through different brainstorming and collective challenges. The results were similar for collaboration (M = 4.75/4.78) during these two techniques.

In addition, the Friedman’s ANOVA and Durbin–Conover post hoc tests indicate that the medians of each activity were not the same. For the multivariate factor, patience, (Chi^2^ (6) = 20.1, *p* = .003) we observed that in the marshmallow challenge, creacapture activity and the creative environment, this factor was chosen by most students, while this was not the case for the land art activity. The test was significant for discipline (Chi^2^ (6) = 12.9, *p* = .044); we found that in activities such as creacapture, the land art and museum activities and the escape game this multivariate factor was more present than in the marshmallow challenge. The results show that the test was significant for the factor, getting organized (Chi^2^ (6) = 13.4, *p* = .037), indicating that during the marshmallow challenge, creative environment and museum activities and the escape game this factor was more present than in the land art activity. The test proposed that the decision factor (Chi^2^ (6) = 14.4, *p* = .025) was observed in the creacapture, world café, land art, creative environment and museum activities and the escape game, but just a few students reported this factor during the marshmallow challenge. For the dynamic factor (Chi^2^ (6) = 13.5, *p* = .036), the results showed that during the world café, land art and museum activities and the escape game this stage was selected more than in the marshmallow challenge. For the collaboration (Chi^2^ (6) = 7.6, *p* = .007) and implication factors (Chi^2^ (6) = 12.7, *p* = .048), the test showed that in activities such as the creacapture, world café, land art, creative environment and museum activities and the escape game this factor was more present than in the marshmallow challenge. Lastly, for multivariate factors such as being kind, perseverance, being a perfectionist, workforce, concentration, quality, discussion and listening the tests were not significant.

#### 7.2.2. Emotional Factors

We also observed the emotional factors in Table 5. We found significant variations for curiosity, surprise, frustration, enthusiasm, awakening, hesitation and stress. For curiosity, the highest score was noted for the museum activity (M = 4.67) and the lowest for the crea-experience (M = 3.31). During the Bionique activity, surprise increased (M = 3.20). The Table 5 shows that frustration was most relevant in the marshmallow challenge (M = 3.22). For enthusiasm the lowest score was observed for the crea-experience (M = 2.13) while the highest was present for the marshmallow challenge and the museum activity (M = 4.22). For awakening the participants gave a higher score during the marshmallow challenge (M = 4.22) and a low score (M = 2.20) during the crea-experience. Hesitation was the most reported factor during the escape game (M = 3.10).

Regarding these factors the result of the Friedman’s ANOVA and post hoc Durbin–Conover tests indicated that the tests were significant for curiosity (Chi^2^ (7) = 25.8, *p* = .001), whereby in the marshmallow challenge, the land art, world café and museum activities, the escape game and the Bionique activity this factor was more present than in the crea-experience. The test was also significant for surprise (Chi^2^ (8) = 19.1, *p* = .014), indicating that during the marshmallow challenge, the creacapture, creative environment and crea-experience activities this factor was mobilized a lot which was not the case for the Bionique activity. In addition, for frustration (Chi^2^ (8) = 17.9, *p* = .022), the test shows that in the creacapture, land art, creative environment museum and Bionique activities, frustration was more present than in the marshmallow challenge. For enthusiasm (Chi^2^ (8) = 18.9, *p* = .016), the test indicates that in the marshmallow challenge, the creacapture, land art, world café, creative environment and museum activities, and the escape game and Bionique activity this factor was chosen much more than in the crea-experience. A similar result was observed for the awakening factor (Chi^2^ (8) = 20, *p* = .010), whereby in the marshmallow challenge, the land art, world café and museum activities and the Escape game and Bionique activity this factor was chosen much more than in the crea-experience.

For the factor, hesitation, the test was significant (Chi^2^ (8) = 20.1, *p* = .010) and presented more during the creacapture, land art, world café, creative environment and museum activities and the crea-experience than in the escape game. For stress (Chi^2^ (8) = 21.8, *p* = .005), the test showed that in the creacapture, land art, world café and creative environment activities this factor was selected much more than in the escape game.

For boredom, anxiety, deception, being proud, inspiration, satisfaction and exhaustion the test was not significant.

### 7.3. Qualitative Data

In addition to the quantitative data from the CDB, we conducted four semi-structured interviews based on evocation (Vermersch 2011; Aden 2016), one and a half years after the training. These interviews helped us to understand what persisted of this creativity course and whether it had an impact on the students’ teaching practices in the long term. We used a thematic analysis by listing the major themes, then we made an inductive and intuitive reading of the interviews in order to observe what the students were really expressing (Paillé and Mucchielli 2016). In this paper we present the social and collaborative dimension expressed by the students.

#### 7.3.1. Interaction with the Group

One of the first memories was the sharing with others as well as the interaction with the group. One student reported: “the advantage was that we were in small groups so we had this opportunity to exchange well and that was an advantage too… what I also remember was the discussions that allowed us to compare our reactions a little”. For one student the group was seen as an activator: “In a good group that was important because it made everyone show their stuff and in the end it worked very well. At the beginning I would say a shy one and then quite enthusiastic”. He also added: “We really saw what everyone was thinking, and then we put together each part of the group’s thought”. These verbatims also show that individuality is one of the important traits for collaboration. Fostering individuality in groups can promote alternate points of view.

Moreover, another participant reported: “there were interactions, I remember a little bit of reactions, or enthusiasm, or a little bit of excitement”, or during the land art activity: “And then, I remember the interactions there was, and I remember that we had built this thing and in the end, it wasn’t really land art, because we had put on… and then you came in and you said it wasn’t really land art because we put on clothes and stuff, so it was kind of funny, that’s what made it stand out”. It seems that this kind of interaction can influence a group and individual satisfaction by creating a good ambience.

Some students emphasized how important it was to be in a good group: “a nice group that worked well, everyone was involved“, or, “we were a good team that participated well”. Some of them mentioned that the change of group encouraged dynamics: “That was interesting because I was not at all with the same people the first and second time during these two challenges, but I felt much more comfortable the second time with the people I was with.”

#### 7.3.2. Role of the Environment

The importance of the environment was described by these verbatims: “But discovering this place, uh interactive offices, for me it was something that was also striking because it is not a universe that we know in our academic world. We probably know it more and more in the world of entrepreneurship”, or even further, “The environment, the fact that we ate there, it wasn’t just that we were trying to do something, but it was embedded in a whole universe. It’s like if you want to do a class on a theater stage and with costumes, it’s pretty much the same thing”. The fact that it was important to have the lessons in different environments was observed in this statement: “(…) it’s important because it allowed you to completely erase what was there before and pass on something completely new each time. And each time, we would reset completely”. Another student reported, “it was the way we were put in context to test the different approaches”.

#### 7.3.3. Difficulties in Group Work

Nevertheless, some participants had a critical stance towards the proposed activities, which was expressed through difficulties in collaboration, especially at the beginning of the activities: “The first time, I had completely faded away because I saw that they were going off in all directions, then there were one or two who were trying to take the lead and trying to really … wanted to give their idea without necessarily making compromises”, or, “I think we had thought too much, we hadn’t experimented and so it was the fact of being in a group, we can’t necessarily, we didn’t hurt…well, I had the impression that we had lost time to finally discuss, what, to understand instead of saying and getting into it, so that’s why it was frustrating”. In the situation where two students had to cooperate with the aim of making a funny presentation during the museum activity, one of them wrote: “it was a bit difficult…but it’s true that we are not necessarily the same personality, so I thought it was someone who was quite... it’s someone who marked me enough”.

According to these verbatims based on the social and collaborative dimensions, we can notice that the participants were in general enthusiastic when they were invited to collaborate and co-create together. Sharing experiences, ideas and knowledge seems to be important for activities in a group, as well as the work environment. It is also evident that collaboration depends on group members and can to some extent create difficulties when participating in some activities.

## 8. Discussion and Conclusions

The results show that the different microlevels of the creative process are subject to variation from one creative experience to another. The same observation is related to the collaborative aspects. Moreover, the CRD allowed us to notice that the creative process is not linear: lots of levels appeared at the same time (definition, questioning, association, experimentation, etc.). Most factors of the multivariate approach emerged from such a creative course. The discussion will allow us to come back both to the research results but also to show the importance of collaboration in the creative process.

### 8.1. Stages of the Creative Process

Regarding the microlevels of the creative process, these may be related to the nature of the task. By testing the variations between the activities of each stage, the test was significant for definition, questioning, illumination, evaluation and finishing.

Many participants reported a problem definition stage during the activities where they had to define the problems in relation to the school through various brainstorming sessions. That was not the case during the activities where the focus was on a challenge or the environment. The definition stage serves to discover the theme and to focus on the subject. This stage corresponds to the preparation stage in Wallas’ model (1926). According to him, it is a preliminary analysis which allows us to define and pose the problem. The preparation stage in both Botella (2011) and Mumford et al.’s (1991) models is crucial for the creative process as it involves acquiring knowledge and expertise. While both models share the preparation stage, the main difference is in the middle stages. Mumford’s model includes incubation and insight, which Botella’s model combines into a single stage of generation (creating ideas and alternatives). In the context of our creative course, this stage was supported using brainstorming sessions which provided an opportunity for the students to gather information, research and develop expertise on a particular topic. By doing so, students are better equipped to generate a wide range of ideas and alternatives during the subsequent stages of the creative process.

The levels of questioning, illumination and evaluation were chosen in a large number of activities by the students. Questioning corresponds to understanding and reflection, which refers to the development of ideas. It seems that in the activities where the students had to propose different solutions or solution tracks these levels were more significant (Botella 2011). This process of generating new ideas or solutions to problems though collaboration with others can encourage individuals to contribute their ideas and perspectives. In collaborative creativity, team members need to search their memory for knowledge or ideas relevant to the problem, share these ideas and process them for further elaboration (Paulus et al. 2018). However, most student did not report these levels in the crea-experience. This may be related to the nature of this activity which did not consist in solving problems. The students performed the same task (summarizing texts) in the two different environments (relaxed versus hostile) during this activity.

In Mumford’s model, the evaluation stage involves critically assessing the idea or solution generated during the insight stage. This assessment may involve considering the potential strengths and weaknesses of the idea or solution and identifying any areas for improvement or refinement. However, Mumford’s model does not include a separate stage for selecting the best idea or alternative. This is the case in Botella’s model. Her model provides a more explicit framework for selecting the most adaptable idea or alternative, while Mumford’s model focuses more broadly on assessing and refining the idea or solution generated. In our creative course, at the end of the idea generation process, the students shared their proposals and evaluated the proposed solutions together. They then selected the solution that was most suitable for the school context.

We can confirm that most of the levels emerged in the students’ creative processes.

### 8.2. Multivariate Factors

Variations were present for some multivariate factors, such as patience, discipline, getting organized, decision-making, dynamics, collaboration and implication. Implication was important during the technique for proposing the generation of ideas in a creative environment, as well as in the activity at the museum and in the escape game. Based on the motivational dynamics proposed by Viau (2009), it originates mainly in the perceptions that a student has of the proposed educational activity.

Variations were also present for some emotional factors, such as curiosity, surprise, frustration, enthusiasm, awakening, hesitation and stress. It is interesting to notice that curiosity and surprise were more significant in activities where the students were generating new ideas or solutions to problems through collaboration with others. In this sense, collaborative creativity can be an effective way to generate a diversity of ideas and perspectives, and can lead to more innovative solutions than would be possible through individual efforts. However, these activities can cause stress and frustration. We suppose that this may be related to the fact that students do not always come up with enough ideas during the brainstorming sessions. We can confirm our hypothesis suggesting that different multivariate factors emerge during the creative process.

Regarding environmental factors, implication and collaboration were most relevant in the creative environment and the museum activity. These creative techniques required idea generation through different brainstorming and collective challenges. Paulus et al. (2016) suggests that group and collaborative creativity refers to the generation of new and useful ideas or products by two or more persons who deliberately engage in a creative/idea generation task. When students work together on a project or task, they are exposed to different perspectives and ideas, which can inspire and stimulate their own creativity. Collaboration also allows students to bounce ideas off each other and work together to solve problems, which can lead to the development of new and innovative solutions. The ability to cooperate is essential because it develops reflexivity and creativity, thanks to the problematization process at work in the cooperative group, a learning organization (Perraud 2019). Furthermore, Marková (2003) shows that exchanges and external and internal dialogues are co-constitutive, and does not reduce groups to the individual, but precisely recognizes the social and dialogical nature of the individual mind (Glăveanu et al. 2021).

The limitation of this pilot research is certainly the sample size (10 participants). While the results of the quantitative analyses are encouraging, caution should be exercised regarding the potential generalizability of the results; a larger sample replication is needed to support these preliminary results.

Despite these limitations, this study provided valuable insights into students’ creative processes. Specifically, it enabled us to identify several distinct stages involved in the process in the creative training context. The variations between the activities for each level help us to better understand what multivariate factors come into play in the creative process.

However, our goal was to create a supportive and open-minded group culture that facilitates collective creativity and encourages the flow of ideas, inviting all members to contribute. Structured creative techniques such as a brainstorming or design thinking adapted to the pedagogical context can be helpful in guiding the creative process and ensuring that all ideas are given equal consideration. This approach can also be embedded in the pedagogy of creativity with the aim of thinking through tasks differently (Puozzo Capron 2013). This also means that, instead of individual activities that stimulate thinking skills, it is important to create opportunities for collaboration and interaction with material and cultural artefacts. According to Dewey’s (1902) research cited in Glăveanu et al. (2015a), this view emphasizes teacher–student collaboration and the grounding of education in everyday life practices, allowing students to relate new knowledge to prior experiences and make use of it in practice. It can deepen their understanding of the world and the possibilities of acting within it. The transformative potential of rethinking pedagogy from a new perspective, however, enables us to transcend traditional educational models and concentrate on dialogical experiences, playful engagement with educational content and a rethink of existence and materiality (Glăveanu et al. 2015b). Making the students aware of the importance of creativity may lead them to teach creativity and divergent thinking in their future classrooms.

## Figures and Tables

**Table 1:** Description of creative activities and experiences during the semester.

Course 1	Marshmallow challenge (group activity)	Used as an ice breaker where the students need to collaborate to make the biggest and the strongest tower from spaghetti.
Course 2	Creacapture (group activity)	An activity asking students to represent creativity via a video, image, recording, etc.
Course 3	World café (group activity)	The students were invited to propose ideas, share knowledge and debate around three topics during three rounds. The activity ended with a presentation of the solutions and main conclusions by each group.
Course 4	Land art (group activity)	The students had to create a “work” from elements found in nature. At the end of their work, they were encouraged to make links with theoretical elements to describe the creative processes experienced in the group. A presentation of the works and a sharing of these processes was made in class.
Course 5	Creative environment (group activity)	After a visit to a creative, co-working space the students tested ordinary and reverse brainstorming in connection with the creative environment.
Course 6	Museum activity (group activity)	Following several challenges and a visit to exhibitions in a museum, the students had to make a presentation related to the discipline they teach.
Course 7	Escape game (group activity)	This activity asked the students to collaborate in order to solve enigmas related to theoretical content. The game was played in different places at the university.
Course 8	Crea-experience (individual activity)	The students lived experiences in two different environments (one zen and the other more hostile) and had to respond to the task of summarizing a text afterwards.
Course 9	Bionique (individual activity)	Following the observation of nature in which the students were able to express themselves in the form of text, drawing, poetry, etc., they were asked to make analogies with the observed elements and the previously defined subject.

**Table 2:** Significant stages.

Stage	Result	Activities
Problem definition	(Chi^2^ (8) = 16.6, *p* = .035)	World café, creative environment, museum activity and escape game
Questioning	(Chi^2^ (8) = 21.8 *p* = .005)	Marshmallow challenge, creacapture, world café, land art, creative environment, museum activity, escape game and Bionique
Illumination	(Chi^2^ (8) = 20.4, *p* = .009)	Creacapture, world café, land art, creative environment and museum activity
Evaluation	(Chi^2^ (8) = 22.2, *p* = .005)	Marshmallow challenge, creacapture, world café, museum activity, escape game and Bionique
Finishing	(Chi^2^ (8) = 16.9, *p* = .032)	World café, land art, creative environment and museum activity

**Table 3:** Frequency of micro-processes reported by students in their CRD.

	Stages with High Scores	Stages with Low Scores
Marshmallow challenge	Constraint (87.5%) Evaluation (87.5%) Realization (87.5%)	Illumination (25%) Chance (25%)
Creacapture	Constraint (100%) Association (89%) Experimentation (89%)	Documentation (22%) Pause (22%) Abandonment (11%)
World café	Definition (80%) Questioning (80%) Finishing (70%)	Abandonment (10%)
Land art	Questioning (80%) Illumination (80%) Association (100%) Evaluation (90%) Structuring (90%) Realization (100%) Finishing (80%)	Abandonment (11%)
Creative environment	Definition (100%) Questioning (100%) Constraint (100%) Illumination (71%) Structuring (90%)	Pause (0)
Museum activity	Illumination (78%) Association (100%) Experimentation (89%) Chance (89%)	Pause (11%) Abandonment (11%)
Escape game	Definition (80%) Questioning (100%) Documentation (100%) Constraint (100%) Association (100%) Evaluation (90%) Chance (90%)	Finishing (20%) Pause (20%)
Crea-experience	Realization (87.5%)	Definition (37%) Questioning (12.5%) Illumination (0) Experimentation (25%) Evaluation (25%) Chance (12.5%) Finishing (25%)
Bionique	Definition (90%) Documentation (80%) Association (90%) Experimentation (90%) Structuring (80%) Chance (90%)	Finishing (30%) Abandonment (20%)

**Table 4:** Significant multivariate factors.

Multivariate Factor	Result	Activities
Patience	Chi^2^ (6) = 20.1, *p* = .003	Marshmallow challenge, creacapture, creative environment
Discipline	Chi^2^ (6) = 12.9, *p* = .044	Creacapture, land art, museum activity, escape game
Getting organized	Chi^2^ (6) = 13.4, *p* = .037	Marshmallow challenge, creative environment, museum activity, escape game
Decision-making	Chi^2^ (6) = 14.4, *p* = .025	Creacapture, world café, land art, creative environment, museum activity, escape game
Dynamics	Chi^2^ (6) = 13.5, *p* = .036	World café, land art, museum activity, escape game
Collaboration	Chi^2^ (6) = 7.6, *p* = .007	Creacapture, world café, land art, creative environment, museum activity, escape game
Implication	Chi^2^ (6) = 12.7, *p* = .048	Creacapture, world café, land art, creative environment, museum activity, escape game

**Table 5:** Significant emotional factors.

Emotional Factors	Result	Activities
Curiosity	Chi^2^ (7) = 25.8, *p* = .001	Marshmallow challenge, land art, world café, museum activity, escape game, Bionique
Surprise	Chi^2^ (8) = 19.1, *p* = .014	Marshmallow challenge, creacapture, creative environment, crea-experience
Frustration	Chi^2^ (8) = 17.9, *p* = .022	Creacapture, land art, creative environment, museum activity, Bionique
Enthusiasm	Chi^2^ (8) = 18.9, *p* = .016	Marshmallow challenge, creacapture, land art, world café, creative environment, museum activity, escape game, Bionique
Awakening	Chi^2^ (8) = 20, *p* = .010	Marshmallow challenge, land art, world café, museum activity, escape game, Bionique
Hesitation	Chi^2^ (8) = 20.1, *p* = .010	Creacapture, land art, world café, creative environment, museum activity, crea-experience
Stress	Chi^2^ (8) = 21.8, *p* = .005	Creacapture, land art, world café, creative environment

## Data Availability

The data presented in this study are available on request from the corresponding author.

## References

[B1-jintelligence-11-00083] Aden Joëlle (2016). L’éducation artistique et culturelle. Enjeux épistémologiques et enjeux politiques de l’évaluation. https://ecp.univ-lyon2.fr/medias/fichier/evaluation-des-activites-artistiques-et-culturelles-rapport-final_1617540032822-pdf.

[B2-jintelligence-11-00083] Amabile Teresa M. (1983). The social psychology of creativity: A componential conceptualization. Journal of Personality and Social Psychology.

[B3-jintelligence-11-00083] Amabile Teresa M. (1996). Creativity in Context: Updates to The Social Psychology of Creativity.

[B4-jintelligence-11-00083] Amabile Teresa M., Pratt Michael G. (2016). The dynamic componential model of creativity and innovation in organizations: Making progress, making meaning. Research in Organizational Behavior.

[B5-jintelligence-11-00083] Amabile Teresa M., Colins Marry A., Conti Regina, Phillips Elise, Picariello Martha, Ruscio John, Dean Whitney (1996). Creativity in Context.

[B6-jintelligence-11-00083] Aragon Cecilia R., Williams Alison (2011). Collaborative creativity: A complex systems model with distributed affect. Paper presented at SIGCHI Conference on Human Factors in Computing Systems.

[B7-jintelligence-11-00083] Audrin Catherine, Vuichard Aleksandra, Capron Puozzo Isabelle (2020). Émotions épistemiques et créativité dans la formation enseignante: Un duo gagnant?. Recherche En Éducation.

[B8-jintelligence-11-00083] Barbier René (1996). La Recherche-Action.

[B9-jintelligence-11-00083] Baudrit Alain (2005). Apprentissage coopératif et entraide à l’école. Revue française de pédagogie.

[B10-jintelligence-11-00083] Beghetto Ronald A., Corazza Giovanni E. (2019). Dynamic Perspectives on Creativity.

[B11-jintelligence-11-00083] Besançon Maud, Georgsdottir Asta, Lubart Todd (2005). La créativité, son développement et l’école. Diversité Veille École Intégration.

[B12-jintelligence-11-00083] Bonnardel Nathalie, Didier John (2016). Enhancing creativity in the educational design context: An exploration of the effects of design project-oriented methods on students’ evocation processes and creative output. Journal of Cognitive Education and Psychology.

[B13-jintelligence-11-00083] Botella Marion (2011). Le Processus Créatif et les émotions: Traits émotionnels et Dynamique Processus-états dans la créativité Artistique [Thèse de doctorat].

[B14-jintelligence-11-00083] Botella Marion, Didier John (2016). Listing the stages of the creative process with teachers of creative activity. International Journal of Psycology.

[B15-jintelligence-11-00083] Botella Marion, Lubart Todd, Beghetto Ronald A., Corazza Giovannni E. (2019). From Dynamic Processes to a Dynamic Creative Process. Dynamic Perspectives on Creativity: New Directions for Theory, Research, and Practice in Education.

[B16-jintelligence-11-00083] Botella Marion, Zenasni Franck, Lubart Todd (2011). A Dynamic and Ecological Approach to the Artistic Creative Process of Arts Students: An Empirical Contribution. Empirical Studies of the Arts.

[B17-jintelligence-11-00083] Botella Marion, Nelson Julien, Zenasni Franck, Capron Puozzo Isabelle (2016). Les macro et microprocessus créatifs. La créativité en éducation et en Formation. Perspectives Théoriques et Pratiques.

[B18-jintelligence-11-00083] Botella Marion, Nelson Julien, Zenasni Franck (2019). It Is Time to Observe the Creative Process: How to Use a Creative Process Report Diary (CRD). Journal of Creative Behavior.

[B19-jintelligence-11-00083] Capron Puozzo Isabelle, Audrin Catherine (2021). Improving self-efficacy and creative self-efficacy to foster creativity and learning in schools. Thinking Skills and Creativity.

[B20-jintelligence-11-00083] Capron Puozzo Isabelle, Botella Marion, Berdal-Masuy Frannçoise (2018). Emo-tissage et créativité. Emo-Tissage.

[B21-jintelligence-11-00083] Capron Puozzo Isabelle, Lapaire Jean-Rémi, Duval Hélène, Tortochot Nathalie Rezzi, Terrien Pascal (2019). Créer et performer pour comprendre: Éléments de théorie et exemples de formation dans des disciplines non-artistiques. Créer pour éduquer. La place de la Transdisciplinarité.

[B22-jintelligence-11-00083] Chappell Kerry (2008). Towards humanising creativity?. UNESCO Observatory: Journal of Multi-Disciplinary Research in the Arts.

[B23-jintelligence-11-00083] Chappell Kerry, Craft Ana, Rolfe Linda, Jobbins Veronica (2012). Humanising Creativity: Valuing our journeys of becoming. International Journal of Education and the Arts.

[B24-jintelligence-11-00083] Corazza Giovannni E. (2016). Potential Originality and Effectiveness: The Dynamic Definition of Creativity. Creativity Research Journal.

[B25-jintelligence-11-00083] Craft Anna (2005). Creativity in Schools: Tensions and Dilemmas.

[B26-jintelligence-11-00083] Cropley David, Cropley Arthur (2012). A psychological taxonomy of organizational innovation: Resolving the paradoxes. Creativity Research Journal.

[B27-jintelligence-11-00083] Csikszentmihalyi Mihaly (1996). Creativity: Flow and the Psychology of Discovery and Invention.

[B28-jintelligence-11-00083] Davis Mark A. (2009). Understanding the relationship between mood and creativity: A meta-analysis. Organizational Behavior and Human Decision Processes.

[B29-jintelligence-11-00083] Dewey John (1902). The school as social center. The Elementary School Teacher.

[B30-jintelligence-11-00083] Didier John, Botella Marion, Attanasio Rachel, Lambert Marie-Dominique, Joël Bisault, Roselyn Le Bourgeois, Jean-François Thémines, Mickaël Le Mentec, Céline Chauvet-Chanoine (2022). Creating a Sound Garden: Transforming Recycled Materials into Objects for Learning. Objects to Learn About and Objects for Learning 2 Which Teaching Practices for Which Issues 11.

[B31-jintelligence-11-00083] Dodge Kenneth A., Perlmutter Marion (1986). A social information processing model of social competence in children. Minnesota Symposium in Child Psychology.

[B32-jintelligence-11-00083] Dul Jan, Ceylan Canan, Jaspers Ferdinand (2011). Knowledge workers’ creativity and the role of the physical work environment. Human Resource Management.

[B33-jintelligence-11-00083] Fischer Gerhar, Vassen Florian (2011). Collective Creativity: Collaborative Work in the Sciences, Literature and the Arts.

[B34-jintelligence-11-00083] Fischer Gerhard, Giaccardi Elisa, Eden Hal, Sugimoto Masanori, Ye Yunwen (2005). Beyond binary choices: Integrating individual and social creativity. International Journal of Human-Computer Studies.

[B35-jintelligence-11-00083] Ghiglione Rodolphe, Matalon Benjamin (1992). Les Enquêtes Sociologiques: Théories et Pratiques.

[B36-jintelligence-11-00083] Glăveanu Vlad P. (2010). Paradigms in the study of creativity: Introducing the perspective of cultural psychology. New Ideas in Psychology.

[B37-jintelligence-11-00083] Glăveanu Vlad-Petre (2011). How are we creative together? Comparing sociocognitive and sociocultural answers. Theory & Psychology.

[B38-jintelligence-11-00083] Glăveanu Vlad-Petre (2021). Creativity: A Very Short Introduction.

[B39-jintelligence-11-00083] Glăveanu Vlad P., Sierra Z., Tanggaard Lene (2015a). Widening our understanding of creative pedagogy: A North–South dialogue. Education.

[B40-jintelligence-11-00083] Glăveanu Vlad, Gillespie Alex, Valsiner Jaan (2015b). Rethinking Creativity: Contributions from Cultural Psychology.

[B41-jintelligence-11-00083] Glăveanu Vlad, Len Tanggaard, Charlotte Wegener (2016). Creativity, a New Vocabulary.

[B42-jintelligence-11-00083] Glăveanu Vlad-Petre, Ness Ingun J., Wasson Barbara, Lubart Tood (2019). Sociocultural perspectives on creativity, learning, and technology. Creativity under Duress in Education? Resistive Theories, Practices, and Actions.

[B43-jintelligence-11-00083] Glaveanu Vlad-Petre, Ness Ingunn J., Rasmussen Ludvig. J. T., McKay Alexander S., Reiter-Palmon Roni, Kaufman James. C. (2021). Creative success in collaboration: A sociocultural perspective. Creative Success in Teams.

[B44-jintelligence-11-00083] Guerra Monica, Villa Federic, Beghetto Ronald A., Corazza Giovanni E. (2019). Exploration as a Dynamic Strategy of Research-Education for Creativity in Schools. Dynamic Perspectives on Creativity New Directions for Theory, Research, and Practice in Education.

[B45-jintelligence-11-00083] Guilford Joy Paul (1950). Creativity. American Psychologist.

[B46-jintelligence-11-00083] Harrington David M. (1999). Conditions and settings/environment. Encyclopedia of Creativity.

[B47-jintelligence-11-00083] Hennessey Beth A. (2003). The social psychology of creativity. Scandinavian Journal of Educational Research.

[B48-jintelligence-11-00083] Johnson David W., Johnson Roger T. (1989). Cooperation and Competition: Theory and Research.

[B49-jintelligence-11-00083] Johnson David W., Johnson Roger T. (2009). An Educational Psychology Success Story: Social Interdependence Theory and Cooperative Learning. Educational Researcher.

[B50-jintelligence-11-00083] John-Steiner Vera (2000). Creative Collaboration.

[B51-jintelligence-11-00083] Kaufman James C., Beghetto Ronald A. (2009). Beyond Big and Little: The Four C Model of Creativity. Review of General Psychology.

[B52-jintelligence-11-00083] Lubart Todd (2001). Models of the Creative Process: Past, Present and Future. Creativity Research Journal.

[B53-jintelligence-11-00083] Lubart Todd (2017). The 7 C’s of Creativity. The Journal of Creative Behavior.

[B54-jintelligence-11-00083] Lubart Todd, Thornhill-Miller Branden (2019). Creativity: An Overview of the 7C’s of Creative Thought.

[B55-jintelligence-11-00083] Lubart Todd, Mouchiroud Christophe, Tordjman Sylvie, Zenasni Franck (2015). Psychologie de la Créativité.

[B56-jintelligence-11-00083] Mandeville Lucie (1998). Les clés de l’expérience: Un modèle d’apprentissage expérientiel pour la formation et l’intervention en psychologie des relations humaines. INTERACTIONS.

[B57-jintelligence-11-00083] Mandeville Lucie, Lafortune Dans Louise, Deaudelin Colette, Doudin Pierre-André, Martin Daniel (2001). Apprendre par l’expérience: Un modèle applicable à la formation continue. La Formation Continue. De la Réflexion à L’action.

[B58-jintelligence-11-00083] Marcy Richard T., Mumford Michael D. (2007). Social innovation: Enhancing creative performance through causal analysis. Creativity Research Journal.

[B59-jintelligence-11-00083] Marková Ivana (2003). Dialogicality and Social Representations: The Dynamics of Mind.

[B60-jintelligence-11-00083] Mouchiroud Christophe, Bernoussi Aurore (2008). An empirical study of the construct validity of social creativity. Including Special Issue on Creativity.

[B61-jintelligence-11-00083] Mumford Michael D., McIntosh Tristan (2017). Creative thinking processes: The past and the future. The Journal of Creative Behavior.

[B62-jintelligence-11-00083] Mumford Michael D., Mobley Michele I., Reiter-Palmon Roni, Uhlman Charrles E., Doares Lesli M. (1991). Process analytic models of creative capacities. Creativity Research Journal.

[B63-jintelligence-11-00083] Nemeth Charlan J., Ormiston Margaret (2007). Creative idea generation: Harmony versus stimulation. European Journal of Social Psychology.

[B64-jintelligence-11-00083] Osburn Holly K., Mumford Michael D. (2006). Creativity and planning: Training interventions to develop creative problem-solving skills. Creativity Research Journal.

[B65-jintelligence-11-00083] Paillé Pierre, Mucchielli Alex (2016). L’analyse Qualitative en Sciences Humaines et Sociales.

[B66-jintelligence-11-00083] Paulus Paul, Nijstad Bernard, Paulus Paul B., Nijstad Bernard A. (2003). Group creativity: An introduction. Group Creativity: Innovation through Collaboration.

[B67-jintelligence-11-00083] Paulus Paul B., Dzindolet Marry, Kohn Nicholas W., Mumford M. D. (2012). Collaborative Creativity—Group Creativity and Team Innovation. Handbook of Organizational Creativity.

[B68-jintelligence-11-00083] Paulus Paul B., van der Zee Karen I., Kenworthy Jared (2016). Cultural diversity and team creativity. The Palgrave Handbook of Creativity and Culture Research.

[B69-jintelligence-11-00083] Paulus Paul B., Baruah Jonali, Kenworthy Jared. B. (2018). Enhancing collaborative ideation in organizations. Frontiers in Psychology.

[B70-jintelligence-11-00083] Peilloux Aurélien, Botella Marion (2016). Ecological and Dynamical Study of the Creative Process and Affects of Scientific Students Working in Groups. Creativity Research Journal.

[B71-jintelligence-11-00083] Perraud Caroline (2019). Une ingénierie coopérative en Esat: Quand travailler ensemble rend capable de créativité. L’exemple emblématique: Une idée farfelue. La nouvelle revue—Éducation et société inclusives.

[B72-jintelligence-11-00083] Pundt Alexander, McKay Alexander S., Reiter-Palmon Roni, Kaufman James C. (2021). Collectively creating music—Creativity in rock bands. Creative Success in Teams.

[B73-jintelligence-11-00083] Puozzo Capron Isabelle (2013). Pédagogie de la créativité: De l’émotion à l’apprentissage. Éducation et socialisation. Les Cahiers du CERFEE.

[B74-jintelligence-11-00083] Purser Ronald E., Montuori Alfonso (2000). In search of creativity: Beyond individualism and collectivism. Paper presented at Western Academy of Management Conference.

[B75-jintelligence-11-00083] Runco Marc A. (1996). Personal creativity: Definition and developmental issues. New Directions for Child Development.

[B76-jintelligence-11-00083] Runco Marc A., Runco Marc A. (2003). Idea evaluation, divergent thinking, and creativity. Critical Creative Processes.

[B77-jintelligence-11-00083] Sadler-Smith Eugene (2016). Wallas’ four-stage model of the creative process: More than meets the eye ?. Creativity Research Journal.

[B78-jintelligence-11-00083] Sawyer R. Keith (2012). Explaining Creativity: The Science of Human Innovation.

[B79-jintelligence-11-00083] Schmoelz Alexander (2018). Enabling co-creativity through digital storytelling in education. Thinking Skills and Creativity.

[B80-jintelligence-11-00083] Scott Ginamarie, Leritz Lyle E., Mumford Michael D. (2004). The effectiveness of creativity training: A quantitative review. Creativity Research Journal.

[B81-jintelligence-11-00083] Simonton Dean K. (2000). Creativity: Cognitive, personal, developmental, and social aspects. American Psychologist.

[B82-jintelligence-11-00083] Spivack George, Shure Myrna B. (1974). Social Adjustment of Young Children: A Congitive Approach to Solving Real-Life Problems.

[B83-jintelligence-11-00083] Stenning Keith, Schmoelz Alexander, Wren Heather, Stouraitis Elias, Scaltsas Theodor, Alexopoulos Konstantine, Aichhorn Amelie (2016). Socratic dialogue as a teaching and research method for co-creativity?. Digital Culture & Education.

[B84-jintelligence-11-00083] Sternberg Robert J., Lubart Todd I. (1995). Defying the Crowd: Cultivating Creativity in a Culture of Conformity.

[B85-jintelligence-11-00083] Sternberg Robert J., Lubart Todd. I. (1996). Investing in creativity. American Psychologist.

[B86-jintelligence-11-00083] Torrance E. Paul (1972). Torrance Tests of Creative Thinking—Directions Manual and Scoring Guide—Figural Test, Booklet A.

[B87-jintelligence-11-00083] Van der Maren Jean M. (2003). La Recherche Appliquée en Pédagogie.

[B88-jintelligence-11-00083] Vermersch Pierre (2011). L’entretien D’explicitation.

[B89-jintelligence-11-00083] Viau Rolland (2009). La Motivation en Contexte Scolaire.

[B90-jintelligence-11-00083] Vuichard Aleksandra, Capron Puozzo Isabelle, Runtz-Christan Edmée, Coen Pierre F. (2021). Créativité. Collection de Concepts-clés de la Formation des Enseignantes et Enseignants en Suisse Romande et au Tessin.

[B91-jintelligence-11-00083] Walsh Christopher. S., Craft Ana, Chappell Kerry, Koulouris Pavlos (2014). Gameful learning design to foster co-creativity. Proceedings of the International Conference of the Australian Association for Research in Education (AARE) and the New Zealand Association for Research in Education (NZARE): Speaking Back through Research.

[B92-jintelligence-11-00083] Westmeyer Hans (1998). The social construction and psychological assessment of creativity. High Ability Studies.

